# Binding Specificity of Two PBPs in the Yellow Peach Moth *Conogethes punctiferalis* (Guenée)

**DOI:** 10.3389/fphys.2018.00308

**Published:** 2018-04-03

**Authors:** Xing Ge, Tofael Ahmed, Tiantao Zhang, Zhenying Wang, Kanglai He, Shuxiong Bai

**Affiliations:** ^1^State Key Laboratory for Biology of Plant Disease and Insect Pest, Institute of Plant Protection, Chinese Academy of Agricultural Science, Beijing, China; ^2^Department of Plant Protection, Henan Institute of Science and Technology, Xinxiang, China; ^3^Bangladesh Sugarcrop Research Institute, Pabna, Bangladesh

**Keywords:** pheromone binding proteins, *Conogethes punctiferalis*, fluorescence competitive binding assays, molecular docking, qPCR

## Abstract

Pheromone binding proteins (PBPs) play an important role in olfaction of insects by transporting sex pheromones across the sensillum lymph to odorant receptors. To obtain a better understanding of the molecular basis between PBPs and semiochemicals, we have cloned, expressed, and purified two PBPs (CpunPBP2 and CpunPBP5) from the antennae of *Conogethes punctiferalis*. Fluorescence competitive binding assays were used to investigate binding affinities of CpunPBP2 and CpunPBP5 to sex pheromone and volatiles. Results indicate both CpunPBP2 and CpunPBP5 bind sex pheromones *E*10-16:Ald, *Z*10-16:Ald and hexadecanal with higher affinities. In addition, CpunPBP2 and CpunPBP5 also could bind some odorants, such as 1-tetradecanol, trans-caryopyllene, farnesene, and β-farnesene. Homology modeling to predict 3D structure and molecular docking to predict key binding sites were used, to better understand interactions of CpunPBP2 and CpunPBP5 with sex pheromones *E*10-16:Ald and *Z*10-16:Ald. According to the results, Phe9, Phe33, Ser53, and Phe115 were key binding sites predicted for CpunPBP2, as were Ser9, Phe12, Val115, and Arg120 for CpunPBP5. Binding affinities of four mutants of CpunPBP2 and four mutants of CpunPBP5 with the two sex pheromones were investigated by fluorescence competitive binding assays. Results indicate that single nucleotides mutation may affect interactions between PBPs and sex pheromones. Expression levels of CpunPBP2 and CpunPBP5 in different tissues were evaluated using qPCR. Results show that CpunPBP2 and CpunPBP5 were largely amplified in the antennae, with low expression levels in other tissues. CpunPBP2 was expressed mainly in male antennae, whereas CpunPBP5 was expressed mainly in female antennae. These results provide new insights into understanding the recognition between PBPs and ligands.

## Introduction

Insects depend on a well-developed olfactory system to distinguish odorants and sex pheromones. Odorant binding proteins (OBPs), chemosensory proteins (CSPs), odorant receptors (ORs) and odorant degrading enzymes (ODEs) are involved in the selectivity and sensitivity of olfaction (Leal, 2005, 2013; Fan et al., 2011; Ahmed et al., 2014, 2017). OBPs are small, water-soluble proteins identified from the chemosensory organs, that are carriers between the external environment and chemoreceptors (Ishida et al., 2002; Leal, 2013). As a multi-genes family, OBPs usually are divided into PBPs, general odorant binding proteins (GOBPs) and antennal binding proteins (ABPs) in lepidopteran insects, based on their binding affinity with sex pheromone and odorant molecules (Vogt et al., 1991; Krieger et al., 1996). Actually, GOBPs and ABPs in many insect species also play roles in pheromone detection, because some of them were found to be expressed in long trichoid sensilla, which are known as pheromone-sensitive sensilla, and most of the main contributors to the ligand binding pocket are conserved (Feng and Prestwich, 1997; Maibeche-Coisne et al., 1998; Zhou et al., 2009; He et al., 2010; Liu et al., 2012). Surprisingly, some GOBP have higher binding affinities with sex pheromone than PBP (Zhou et al., 2009; Liu et al., 2012). PBPs are thought to bind and transport hydrophobic sex pheromone molecules across the aqueous sensillum-lymph to specific pheromone receptors on the dendritic membrane of olfactory neurons (Vogt and Riddiford, 1981; Leal et al., 2005; Forstner et al., 2006; Pelosi et al., 2006). In the earlier studies, PBPs are considered mostly male-specific, while other OBPs are expressed in both males and females (Pelosi et al., 2006). As the first step of pheromone recognition, when PBPs bind to different components of sex pheromones, they can lead to species specificity (Willett and Harrison, 1999).

So far, the 3D structure of PBPs in *Bombyx mori* (Sandler et al., 2000; Horst et al., 2001), *Antheraea polyphemus* (Mohanty et al., 2004), *Leucophaea maderae* (Lartigue et al., 2003), *Amyelois transitella* (Xu et al., 2010; di Luccio et al., 2013), *Apis mellifera* (Lartigue et al., 2004) have been elucidated both alone and in combination with various ligands. The structure of *B. mori* PBP (BmorPBP) with bombykol was the first to be studied by X-ray diffraction spectroscopy and nuclear magnetic resonance (NMR) techniques (Sandler et al., 2000; Horst et al., 2001). The binding pocket of BmorPBP was formed by four antiparallel helices (α1, α4, α5, and α6; Sandler et al., 2000), and the conformational transition in solution displayed pH-dependence (Horst et al., 2001). Stability of protein and ligands are maintained by amino acid residues. Some of these residues are critical for binding ligands (Sandler et al., 2000; Mohanty et al., 2004; Thode et al., 2008; Jiang et al., 2009; Yin et al., 2015; Tian and Zhang, 2016; Zhu et al., 2016; Ahmed et al., 2017; Zhang et al., 2017). Of the residues in BmorPBP, Met5, Phe12, Phe36, Trp37, Ile52, Ser56, Phe76, Val94, Glu98, Ala115, and Phe118 are more conserved and involved in binding to bombykol, which suggests they are interacting with ligands (Sandler et al., 2000; Klusák et al., 2003). Thr57, Ser52 and Thr48 in *Drosophila melanogaster* LUSH are involved in the binding of short-chain *n*-alcohols. Thr57 mutants had a significant decrease in ability to bind alcohol compounds compared with wild type, which indicates Thr57 is the key site of LUSH binding to small alcohol molecules (Kruse et al., 2003; Thode et al., 2008).

Insect pheromones play an important role in intra-species communication, sexual attraction, mating aggregation and oviposition host-marking. In many moth species, sex pheromones are usually blends of chemical compounds. Airborne pheromones of moths often consist of two or three chemical components, each of which is perceived by specific olfactory receptor neurons (Abraham et al., 2005).

The yellow peach moth, *Conogethes punctiferalis* (Guenée; Lepidoptera: Crambidae), is an important agricultural pest of peach, apple, chestnut, maize, and sorghum (Luo and Honda, 2015; Ge et al., 2016). The main sex pheromone compounds of yellow peach moth are (*E*)-10-hexadecenal (*E*10-16:Ald), along with the two minor components (*Z*)-10-hexadecenal (*Z*10-16:Ald) and hexadecenal (16:Ald; Konno et al., 1982; Liu et al., 1994; Kyungsaeng and Park, 2005). Field trials indicate that *Z*10-16:Ald and 16:Ald alone do not attract males. A blend of these compounds (two or three) was more attractive (Liu et al., 1994). A better understanding of the molecular mechanisms of sex pheromone perception would improve the use of pheromones to control this pest. In this study, two PBP genes, CpunPBP2 and CpunPBP5, which were identified as pheromone binding proteins, are cloned in the antennae of *C. punctiferalis* and successfully expressed in *Escherichia coli*. In order to better understand the function of these PBPs, fluorescence displacement binding assays of CpunPBP2 and CpunPBP5 and their mutants are carried out with sex pheromone components.

## Materials and methods

### Insects rearing

*C. punctiferalis* larvae were collected from the sunflower *Helianthus annuus* at Langfang Experimental Station of Chinese Academy of Agricultural Sciences, Hebei Province, China, and reared on fresh maize in an environmentally controlled room. Rearing conditions were 27 ± 1°C, 70–80% relative humidity (RH) and 16:8 light: dark (L:D). Adults were provided with 10% honey solution. After eclosion, the antennae from males and females (80 pairs of each sex) were immediately cut and processed for RNA extraction.

### RNA extraction and reverse transcription

Total RNA was isolated from the antennae using Trizol Reagent (Invitrogen, Carlsbad, CA, USA) following manufacturer's recommendations. The integrity of total RNA was assessed with 1.2% agarose gel electrophoresis and the concentration was determined on a NanoDrop 2000 spectrophotometer (Thermo, USA). One μg RNA was added for reverse transcription to cDNA according to product kit instructions (TransGen, Beijing, China).

### Cloning and sequencing

CpunPBP2 (GenBank accession number: GEDO010000019.1; Jia et al., 2016) and CpunPBP5 (GenBank accession number KP985227) of *C. punctiferalis* were obtained from the antennal cDNA library. The primers were designed to clone the coding region of CpunPBP2 and CpunPBP5 (Table S1; Underlined bases show restriction enzyme sites for forward and reverse primers, respectively). PCR products were separated by electrophoresis on 1% agarose gels in 1 × TAE buffer. Then the specific fragments were cut and purified by DNA gel extraction kit (Axygen, Hangzhou, China) following the manufacturer's protocol. The purified products were cloned into pGEM-T easy vector (TransGen, Beijing, China) and then transformed to TransT1 *E. coli* competent cells (TransGen, Beijing, China). Positive clones were selected by PCR using M13 primers and then sequenced.

### Sequencing analysis

Sequences obtained for alignment and phylogenetic tree construction were downloaded from NCBI database (https://www.ncbi.nlm.nih.gov/), the putative signal peptides were predicted with SignalP 4.1 server (http://www.cbs.dtu.dk/services/SignalP/). Sequence alignments were produced with DNAMAN software. The phylogenetic tree was constructed using the neighbor-joining method with the MEGA 5.2 program (bootstrapping with 1,000 replications; Tamura et al., 2011). Evolutionary distances were computed using the Poisson correction method.

### Recombinant protein expression and purification

Prokaryotic expression system (Gu et al., 2012) was used to express CpunPBP2 and CpunPBP5. First, the pGEM plasmid containing the positive clones were digested by Bam H*I* and Hind *III* enzymes (NEB, Beijing, China). The expected band was purified and cloned into the bacterial expression vector pET 30a(+) digested with the same enzymes. The pET 30a(+)-CpunPBP2 and pET 30a(+)-CpunPBP5 were transformed into the TransT1 competent cells and grown on LB solid medium with 10 μL kanamycin (10 mg/mL). Positive colonies were selected by PCR using T7 primers and transformed into BL21 (DE3) competent cells (TransGen, Beijing, China). The verified single colony was cultured overnight in 5 mL LB broth including 50 μg/mL kanamycin. LB broth (0.5 L) was inoculated with 5 mL overnight culture at 37°C for 3 h until the absorbance at OD_600_ reached to 0.6. Then the protein was induced with isopropyl-β-d-thiogalactoside (IPTG) in a final concentration of 1 mM at 37°C for 6 h (Prestwich, 1993). The induced bacterial cells were centrifuged at 4°C for 10 min (10,000 rpm) and resuspended in the PBS buffer (NaCl 137 mmol/L, KCl 2.7 mmol/L, Na_2_HPO_4_ 10 mmol/L, KH_2_PO_4_ 2 mmol/L, pH 7.4), agitated by ultrasonic waves (an interval of 5 s, 10 min) and centrifuged again (15,000 rpm, 20 min, 4°C). The supernatant and pellet were analyzed by sodium dodecyl sulfate polyacrylamide gel electrophoresis (SDS-PAGE), which showed that CpunPBP2 and CpunPBP5 were expressed mainly in the precipitate. Precipitate was resolved in 8M carbamide and purified by 6 × His-Tagged Purification Kit (CWbio, Beijing, China). Refolded proteins were dialyzed within PBS buffer overnight at 4°C and then concentrated using Amicom 10 kDa cutoff concentrators (Millipore Billerica, MA, USA). The purity and size were checked by SDS-PAGE. The concentration was determined by the Bradford method using bovine serum albumin (BSA) as standard protein.

### Fluorescence displacement binding assay

Fluorescence binding assay was used to measure the affinity of the CpunPBP2 and CpunPBP5 to 3 sex pheromone and 21 volatile compounds (Konno et al., 1982; Kyungsaeng and Park, 2005). The fluorescence intensity was recorded on a FluoroMax-4 spectrophotometer (Horiba Scientific, USA) at room temperature using a 1 cm light path fluorimeter quartz cuvette. The fluorescent probe N-phenyl-1-naphthylamine (1-NPN) and all the tested chemicals were dissolved in HPLC purity methanol. The final concentration was prepared 1 mM. To measure the affinity of florescent ligand 1-NPN to each protein, a 2 μM solution of the protein in 50 mM Tris-HCl, pH 7.4, was titrated with aliquots of 1 mM ligand in methanol to final concentrations of 1–8 μM. The fluorescence of 1-NPN was excited at 337 nm and emission spectra were recorded between 300 and 450 nm. The affinity of other ligands was measured in competitive binding assays, using 1-NPN as the fluorescent reporter at 2 μM concentration and different concentrations of each ligands. The GraphPad Prism 5 (GraphPad Software, Inc.) was used to estimate the K_1−NPN_ (K_D_ of complex protein /1-NPN) values by nonlinear regression for a unique site of binding. It was assumed that the proteins were 100% active, with a stoichiometry of 1:1 (protein:ligand) at saturation. For other competitor ligands, the dissociation constants were calculated from the corresponding IC_50_ (concentrations of ligands halving the initial fluorescence value of 1-NPN) values using Microsoft Office Excel 2010, with the formula: K_D_ = [IC_50_]/(1+[1-NPN]/K_1−NPN_). In the equation, [1-NPN] is the free concentration of 1-NPN, and K_1−NPN_ is the dissociation constant of the complex protein /1-NPN.

### Molecular docking

Sequences of CpunPBP2 and CpunPBP5 were submitted to the SWISS-MODEL server (http://swissmodel.expasy.org/) for structural modeling with all known proteins to obtain template sequences. Then target and template sequences were aligned with ClustalW program. Finally, three dimensional models of CpunPBP2 and CpunPBP5 were generating using I-TASSER Protein Structure and Function Prediction web server (http://zhanglab.ccmb.med.umich.edu/I-TASSER/; Zhang, 2008; Yang et al., 2015). The 3D structure of *E*10-16:Ald and *Z*10-16:Ald were obtained from ChemOffice (http://www.cambridgesoft.com/Ensemble_for_Chemistry/ChemOffice/ChemOfficeProfessional/) and was further refined by the CHARMm force field (http://www.charmm.org/). The model was rendered in PyMol (http://www.pymol.org/). The energy minimization was used to refine the ligand poses. Based on the established homology model, the docking program CDOCKER was used to dock the sex pheromone compounds (*E*10-16:Ald and *Z*10-16:Ald) with CpunPBP2 and CpunPBP5 models, respectively. The binding energy included van der Waals energy (E_vdw_), electrostatic interaction energy (E_eie_) and total interaction energy (E_total_). The energy required for interactions among sex pheromone and CpunPBP2 and CpunPBP5 were calculated to select key residues.

### Preparation of site-directed mutants

Four mutants of CpunPBP2 and four mutants of CpunPBP5 were developed using the QuikChange Lightning Site-Directed Mutagenesis Kit (Stratagene, USA). The mutational primers were designed manually. Mutation sites are underlined in Table S2. The CpunPBP2/pGEM-T Easy construct was used as a template. The PCR conditions were 95°C for 5 min, followed by 30 cycles of 95°C for 30 s, 58°C for 30 s and 68°C for 1 min, and final extension at 72°C for 10 min. The correct insertion of mutation was subcloned into pGEM-T Easy vector (TransGen, Beijing, China). The expression system and fluorescence binding assay were conducted as mentioned for wild type proteins.

### Relative expression pattern of CpunPBP2 and CpunPBP5

Antennae, proboscises, maxillary palps, thoraxes, legs, abdomens, heads (without antennae, proboscises, and maxillary palps), and wings (50 pairs of each sex) were collected for total RNA extraction using Trizol reagent (Invitrogen, Carlsbad, CA, USA). The first strand cDNA template was synthetized with One-Step gDNA removal and cDNA Synthesis kit (TransGen, Beijing, China) including oligo dt-primer according to product manual recommendations. The primers of CpunPBP2, CpunPBP5 and reference gene (β-actin, accession number JX119014) for real-quantitative PCR (qPCR) were designed using Primer premier 5.0 program (Premier Biosoft International, Palo Alto, CA, USA; Table S1). qPCR were conducted on ABI 7500 fast real-time PCR system (Applied Biosysterm, USA). Each amplification reaction was performed with 20 μL volume using SYBR Premix Ex Taq II (Tli RNaseH Plus) master mix (Takara-Bio, Shiga, Japan) under the following conditions: 95°C for 30 s, followed by 40 cycles of 95°C for 3 s and 60°C for 30 s. To check reproducibility, each test sample was done in triplicate technical replicates and three biological replicates. Relative quantification was analyzed using the comparative 2^−ΔΔCT^ method (Livak and Schmittgen, 2001). The relative expression levels in different tissues were calculated with the transcript level of the female antennae used as the calibrator.

## Results

### Sequence analysis of CpunPBP2 and CpunPBP5

Coding regions of CpunPBP2 and CpunPBP5 were obtained from the antennal cDNA library. Sequence analysis shows that the full-length cDNA encoding CpunPBP2 consists of 513 nucleotides that encode 170 amino acid residues. SignalP predicts that the signal peptide cleavage sites have 25 amino acids. On the other hand, CpunPBP5 contains 507 nucleotides for a polypeptide of 168 amino acids with 25 amino acids as signal peptide. The alignment of amino acid sequences shows that CpunPBP2 and CpunPBP5 have six conserved cysteines, which are typical of classic OBPs (Figure S1; Pelosi et al., 2006). Additionally, a few amino acids also are conserved in the aligned sequences. Compared with the other 81 Lepidopteran PBPs, the phylogenetic tree based on the amino acid sequences shows that CpunPBP2 and CpunPBP5 share closer ancestry with PBPs in Crambidae, Lepidoptera (Figure S2).

### Recombinant protein expression and fluorescence displacement binding assay

**Figure 1:**
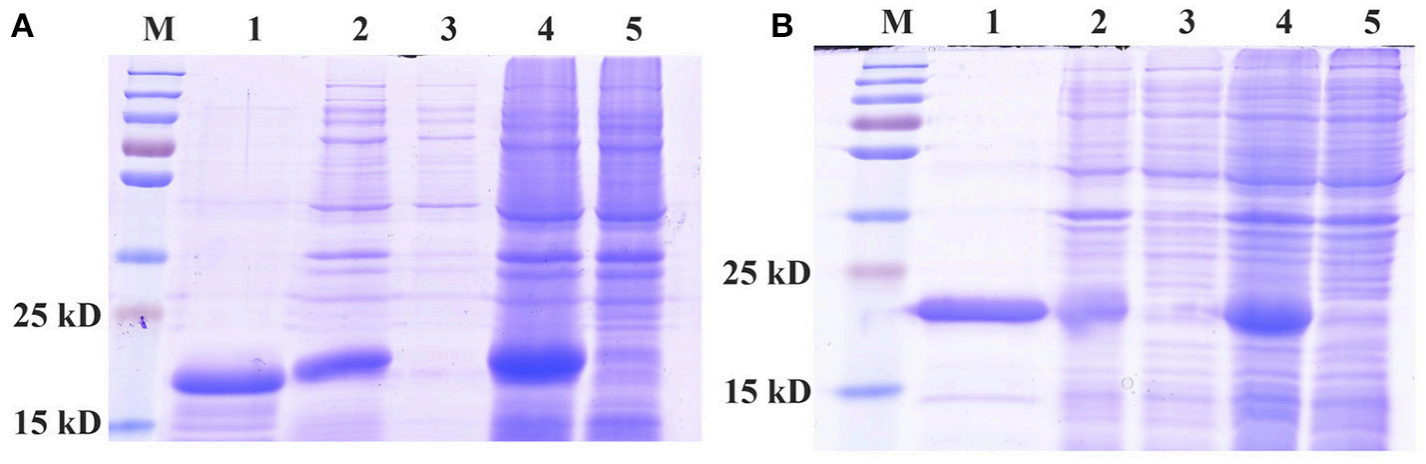
SDS-PAGE analyses of expressed recombinant of CpunPBP2 **(A)** and CpunPBP5 **(B)**. M, marker protein; 1, purified fusion protein; 2, inclusion body of induced cells; 3, supernatant from ultrasound treated cells; 4, IPTG induced *E. coli* pET30a (+)/CpunPBPs transformed BL 21(DE3) cells; 5, Non-induced pET30a (+)/CpunPBPs transformed BL 21(DE3) cells.

Recombinant CpunPBP2 and CpunPBP5 (wild type) proteins expressed in *E. coli* occurred in inclusion bodies and were high yield. The precipitate was resuspended and purified by affinity chromatography (Figure 1) to produce ~1 mg/mL protein, which was used in the fluorescence displacement binding assay.

**Figure 2:**
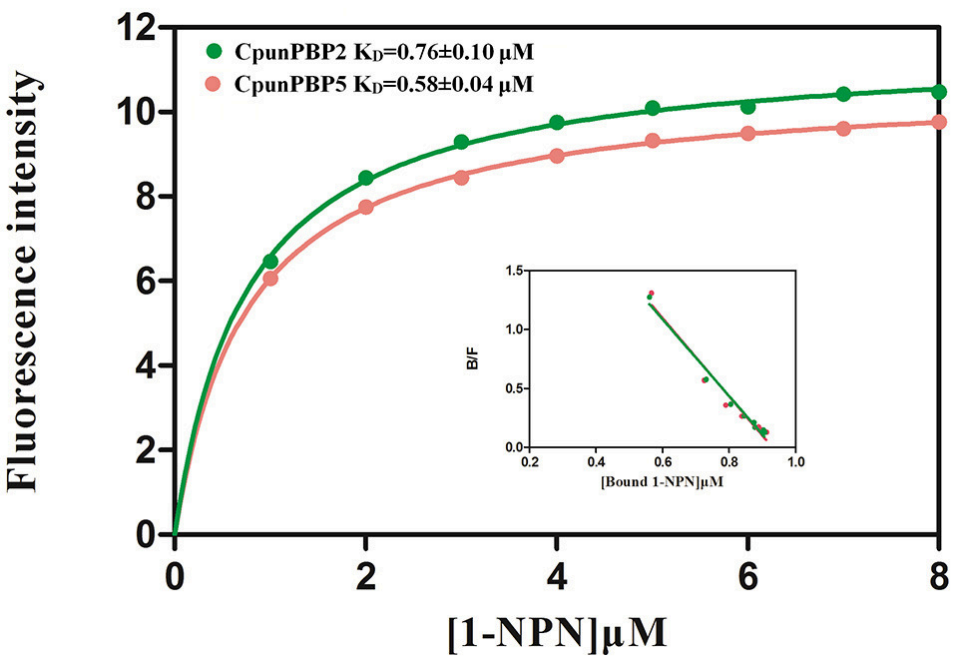
The binding curve and relative Scatchard plots of 1-NPN with CpunPBP2 and CpunPBP5.

Fluorescence of CpunPBP2/1-NPN and CpunPBP5/1-NPN complexes were excited at 337 nm, and the fluorescence peak was 390–410 nm. The dissociation constants (K_D_) of CpunPBP2/1-NPN and CpunPBP5/1-NPN complexes are 0.76 ± 0.10 μM and 0.58 ± 0.04 μM as measured by Scatchard plots (Figure 2). The IC_50_ values and the calculated K_D_ of 21 volatiles and 3 sex pheromone analogs to CpunPBP2 and CpunPBP5 are shown in Table 1.

**Table 1:** IC_50_ values (μM) and calculated dissociation constants (K_D_) (μM) of CpunPBP2 and CpunPBP5 to different ligands at pH = 7.4.

**Ligand**	**CpunPBP2**			**CpunPBP5**		
	**IC_50_ (μM)**	**Int (%)**	**K_D_ (μM)**	**IC_50_ (μM)**	**Int (%)**	**K_D_ (μM)**
**ALDEHYDES**						
Decanal	13.14 ± 0.24	72.86 ± 0.24	7.62 ± 0.14	15.19 ± 0.60	72.60 ± 0.11	12.49 ± 0.49
Heptanal	17.90 ± 0.83	84.85 ± 0.31	10.38 ± 0.48	19.68 ± 0.95	68.32 ± 0.21	15.92 ± 0.77
Undecanal	7.49 ± 0.15	56.59 ± 0.32	4.34 ± 0.08	8.67 ± 0.04	58.66 ± 0.31	7.01 ± 0.03
Trans-2-nonenal	22.25 ± 1.30	86.46 ± 0.38	12.91 ± 0.76	9.62 ± 0.58	58.97 ± 0.62	7.79 ± 0.48
Trans-2-octanal	12.32 ± 0.84	62.32 ± 0.13	7.15 ± 0.49	12.22 ± 0.66	62.12 ± 0.30	9.89 ± 0.53
Nonanal	14.66 ± 0.47	77.22 ± 0.07	8.50 ± 0.27	13.10 ± 0.62	65.83 ± 0.28	10.60 ± 0.78
Hexenal	34.56 ± 0.12	83.11 ± 0.15	20.04 ± 0.68	11.66 ± 0.66	60.86 ± 0.49	9.44 ± 0.53
*Z*10-16:Ald	0.98 ± 0.09	42.51 ± 1.88	0.42 ± 0.04	0.97 ± 0.02	40.58 ± 1.39	0.36 ± 0.01
*E*10-16:Ald	0.94 ± 0.03	39.45 ± 0.83	0.40 ± 0.01	1.33 ± 0.06	39.98 ± 1.53	0.60 ± 0.02
Hexadecanal	1.84 ± 0.02	34.03 ± 0.34	1.07 ± 0.01	4.51 ± 0.08	46.83 ± 0.18	3.65 ± 0.07
Vanillin	19.92 ± 1.00	85.40 ± 0.31	11.56 ± 0.58	25.15 ± 2.90	71.19 ± 0.36	20.36 ± 2.35
**ALCOHOLS**		**PALMITIC ACID**				
Linalool	22.01 ± 4.72	72.27 ± 0.43	12.76 ± 2.74	16.97 ± 1.42	66.92 ± 0.13	13.73 ± 1.15
1-tetrodecanol	3.94 ± 0.14	45.55 ± 0.38	2.28 ± 0.08	3.53 ± 0.03	42.29 ± 0.12	2.86 ± 0.02
cis-3-hexen-1-ol	34.46 ± 3.20	89.59 ± 0.25	19.99 ± 1.85	18.36 ± 1.38	66.81 ± 0.61	14.87 ± 1.12
**OLEFINES**						
α-pinene	27.92 ± 4.30	77.70 ± 0.33	16.20 ± 2.50	14.09 ± 1.26	74.88 ± 0.39	11.41 ± 1.02
β-pinene	21.02 ± 2.26	80.45 ± 0.68	12.19 ± 1.31	11.50 ± 0.39	68.43 ± 0.63	9.3 ± 0.31
Farnesene	4.64 ± 0.24	45.58 ± 0.92	2.69 ± 0.14	4.83 ± 0.03	45.50 ± 0.09	3.91 ± 0.02
β-farnesene	2.82 ± 0.19	40.51 ± 0.78	1.63 ± 0.11	5.25 ± 0.02	47.47 ± 0.12	4.25 ± 0.02
Trans-caryopyllene	6.17 ± 0.14	50.42 ± 0.51	3.58 ± 0.08	4.67 ± 0.02	45.87 ± 0.10	3.78 ± 0.01
Limonene	11.93 ± 0.67	82.86 ± 0.44	6.92 ± 0.39	10.71 ± 0.22	60.13 ± 0.09	8.67 ± 0.18
**OTHERS**						
α-ionone	10.73 ± 0.97	61.77 ± 1.49	6.22 ± 0.56	8.39 ± 0.10	55.28 ± 0.10	6.79 ± 0.08
β-ionone	17.34 ± 0.84	88.04 ± 0.58	10.06 ± 0.49	12.17 ± 0.47	62.62 ± 0.79	9.85 ± 0.38
Palmitic acid	14.25 ± 0.71	73.64 ± 0.06	8.27 ± 0.41	7.27 ± 0.06	53.54 ± 0.01	5.89 ± 0.47
2,6-Dimethyloctane	11.01 ± 0.32	78.17 ± 0.43	6.38 ± 0.19	8.20 ± 0.10	54.82 ± 0.07	6.64 ± 0.08

*The Int represents the ration of fluorescence intensity values at the pheromone concentration of 6 mM to the initial fluorescence intensity without the pheromone*.

*The farnesene is a mixture of α-farnesene and β-farnesene*.

**Figure 3:**
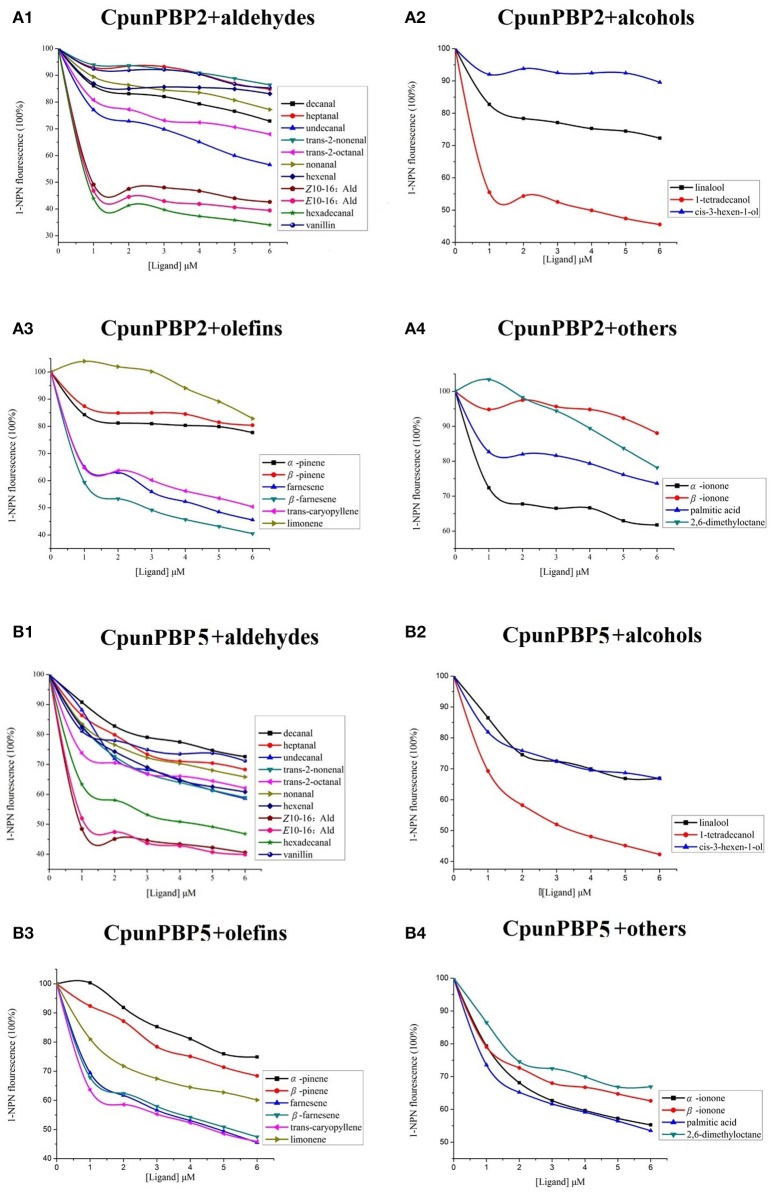
Competitive binding curves of CpunPBP2 and CpunPBP5 to different ligands. **(A1)** Competitive binding curves of CpunPBP2 to aldehydes. **(A2)** Competitive binding curves of CpunPBP2 to alcohols. **(A3)** Competitive binding curves of CpunPBP2 to olefins. **(A4)** Competitive binding curves of CpunPBP2 to other compounds. **(B1)** Competitive binding curves of CpunPBP5 to aldehydes. **(B2)** Competitive binding curves of CpunPBP5 to alcohols. **(B3)** Competitive binding curves of CpunPBP5 to olefins. **(B4)** Competitive binding curves of CpunPBP5 to other compounds.

Fluorescence intensity of CpunPBP2 and CpunPBP5 gradually declined with the increased concentrations of volatiles and sex pheromone (Figure 3). The results show that CpunPBP2 and CpunPBP5 have the highest binding ability to sex pheromones *E*10-16:Ald, *Z*10-16:Ald compared with hexadecanal and other volatiles. Compared with CpunPBP5, CpunPBP2 has a higher binding affinity to the sex pheromone *E*10-16:Ald and hexadecanal. CpunPBP2 also has a similar binding affinity between *E*10-16:Ald and *Z*10-16:Ald. This result indicates there is a definite apparent interaction between the sex pheromones and the two PBPs. Among the volatiles, the binding results indicate that 1-tetradecanol had the highest binding affinity with CpunPBP2 and CpunPBP5, followed by trans-caryopyllene, farnesene, β-farnesene. Interestingly, results also indicate that CpunPBP2 and CpunPBP5 could discriminate the chiral structure of chemical molecules. The two PBPs could bind to α-ionone better than its isomer β-ionone, while is counter to the isomer of pinene. Hexenal, cis-3-hexen-1-ol, α-pinene, β-pinene, trans-2-nonenal and linalool had the minimum binding affinities to CpunPBP2. The vanillin, heptanal, cis-3-hexen-1-ol, linalool had the minimum binding abilities to CpunPBP5.

### Molecular docking

**Figure 4:**
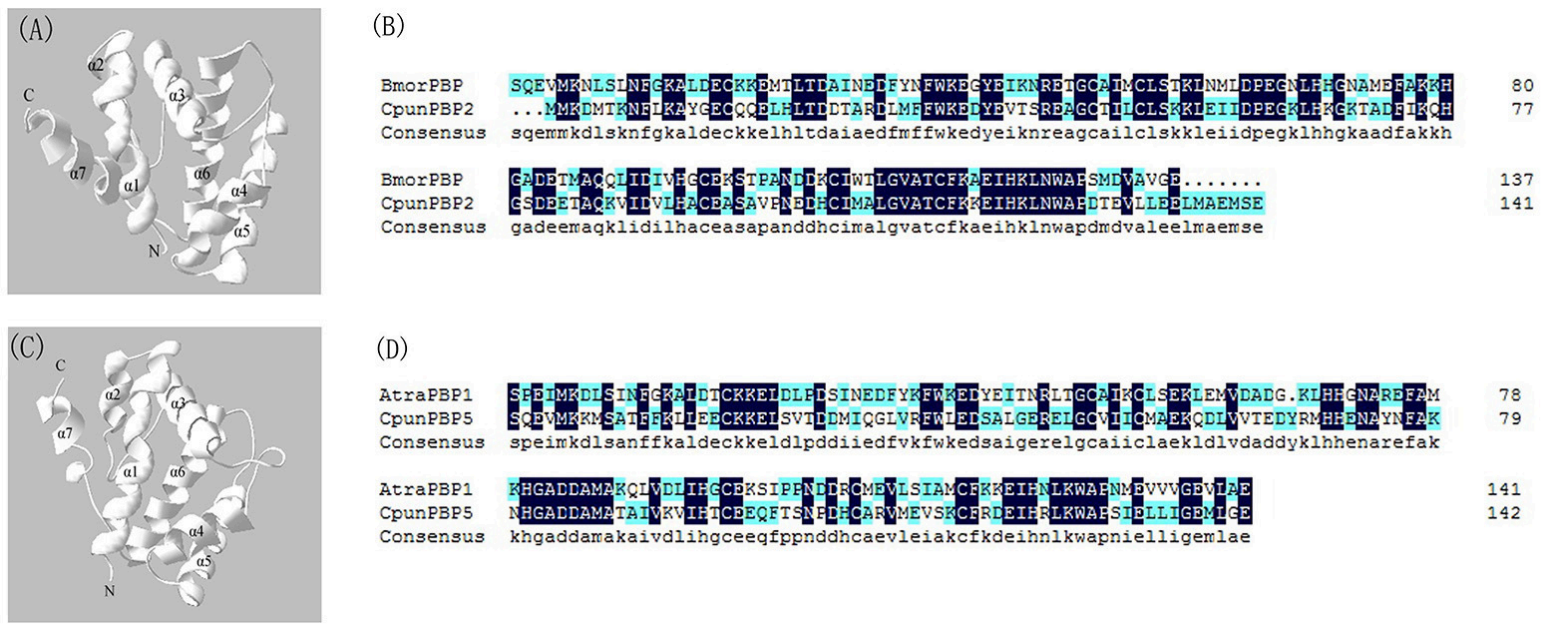
3D structure model of the CpunPBP2 and CpunPBP5. **(A)** Predicted 3D model of CpunPBP2 was built on structure of PBP1 from *Bombyx mori*. Seven α-helixes, N-terminal (Nt) and C-terminal (Ct) are marked. **(B)** Sequence alignment of CpunPBP2 and BmorPBP. In the alignment of the two proteins, identical residues are highlighted in blue. **(C)** Predicted 3D model of CpunPBP5 was built on structure of PBP1 from *Amyelois transitella*. Seven α-helixes, N-terminal (Nt) and C-terminal (Ct) are marked. **(D)** Sequence alignment of CpunPBP5 and AtraPBP1. In the alignment of the two proteins, identical residues are highlighted in blue.

**Figure 5:**
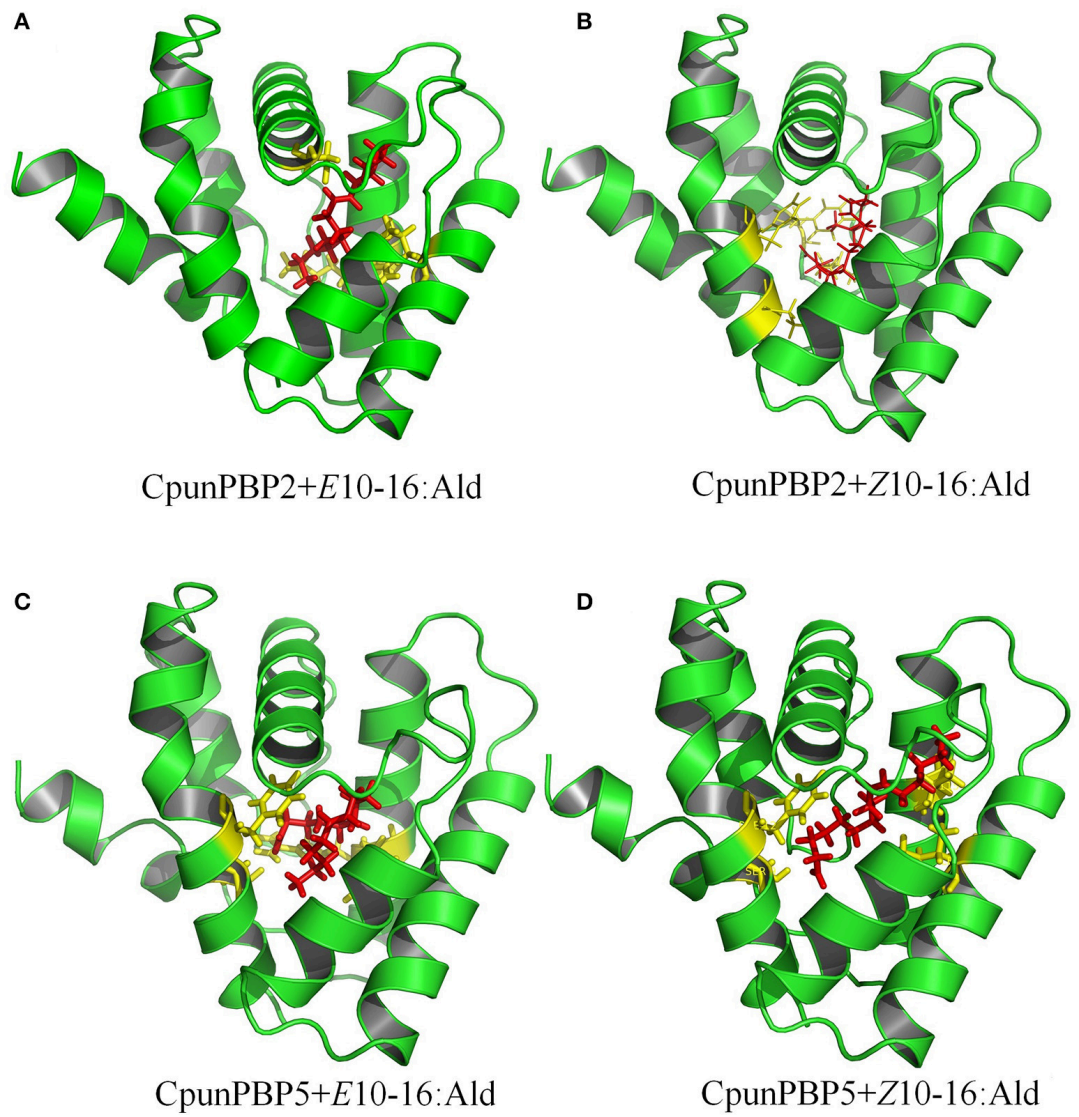
Docking of CpunPBP2 and CpunPBP5 with pheromone compounds. **(A)** Molecular docking of predicted CpunPBP2 model with *E*10-16:Ald. **(B)** Molecular docking of predicted CpunPBP2 model with *Z*10-16:Ald. **(C)** Molecular docking of predicted CpunPBP5 model with *E*10-16:Ald. **(D)** Molecular docking of predicted CpunPBP5 model with *Z*10-16:Ald.

To predict the 3D structure of CpunPBP2 and CpunPBP5, sequences from other similar proteins were compared. The search suggests BmorPBP (PDB id: 1ls8) and AtraPBP1 (PDB id: 2 kph) were used to construct the 3D structure of CpunPBP2 and CpunPBP5 with high similarity (54.0 and 45.8%), respectively (Figure 4). The predicted 3D structure of CpunPBP2 and CpunPBP5 consists of seven α-helices, and the antiparallel helices converge to form the hydrophobic binding pocket (Figure 4). To further study the binding site of sex pheromone to CpunPBP2 and CpunPBP5, *E*10-16:Ald and *Z*10-16:Ald were docked with the predicted CpunPBP2 and CpunPBP5 models (Figure 5). The interaction energies between key residues and the ligands are predicted and listed in Tables 2, 3. Based on the interaction energy of docking models, several residues including Phe9, Phe33, Ser53, and Phe115 in CpunPBP2 and Ser9, Phe12, Val115, and Arg120 in CpunPBP5 seem to play crucial roles in the binding to *E*10-16:Ald and *Z*10-16:Ald.

**Table 2:** Interaction energies (k_cal_/mol) between the key residues of CpunPBP2 and pheromone compounds.

**CpunPBP2**	***E*****10-16:Ald**			***Z*****10-16:Ald**		
	**E_total_**	**E_vdw_**	**E_eie_**	**E_total_**	**E_vdw_**	**E_eie_**
MET2	−1.393	−1.223	−0.170	−1.240	−1.171	−0.069
MET5	–	–	–	−2.290	−2.239	−0.051
THR6	−0.783	−0.885	0.102	−1.918	−2.129	0.211
PHE9	−1.363	−1.515	0.152	−4.469	−4.054	−0.415
PHE33	−1.235	−1.245	0.010	−3.016	−1.959	−1.058
TRP34	−0.741	−1.942	1.202	−0.915	−1.753	0.838
ILE49	−1.716	−1.714	−0.002	−2.149	−2.134	−0.015
LEU50	−1.136	−1.125	−0.011	–	–	–
LEU52	–	–	–	0.027	−0.517	0.543
SER53	−2.729	−2.640	−0.089	−1.746	−1.175	−0.571
LEU56	−0.182	−0.215	0.033	−0.493	−0.539	0.045
ILE58	−1.196	−1.287	0.091	−1.874	−2.004	0.130
GLY63	−0.557	−0.534	−0.022	–	–	–
LEU65	−2.034	−2.105	0.071	−0.596	−0.604	0.008
THR70	–	–	–	−1.290	−1.671	0.380
VAL87	–	–	–	−0.881	−0.644	−0.237
LEU91	−2.718	−2.857	0.140	−2.109	−2.050	−0.059
ALA108	−1.804	−1.935	0.132	−0.180	−0.423	0.243
VAL111	−1.260	−1.553	0.293	–	–	–
ALA112	−1.278	−1.637	0.359	−2.340	−2.353	0.014
PHE115	−4.134	−3.944	−0.190	−2.088	−2.349	0.261
ILE119	−1.474	−1.236	−0.237	–	–	–
LEU131	−0.308	−0.268	−0.040	−1.786	−1.501	−0.285

*E_total_, total interaction energy; E_vdw_, Van der Waals energy; E_eie_, electrostatic interaction energy*.

**Table 3:** Interaction energies (k_cal_/mol) between the key residues of CpunPBP5 and pheromone compounds.

**CpunPBP5**	***E*****10-16:Ald**			***Z*****10-16:Ald**		
	**E_total_**	**E_vdw_**	**E_eie_**	**E_total_**	**E_vdw_**	**E_eie_**
MET5	−0.764	−1.058	0.294	−0.997	−1.144	0.146
MET8	−2.308	−1.781	−0.527	−0.715	−0.850	0.135
SER9	−10.204	−1.951	−8.253	−7.416	−2.154	−5.262
PHE12	−5.351	−4.785	−0.566	−3.593	−3.777	0.185
PHE13	−0.862	−0.553	−0.309	–	–	–
LEU33	−0.273	−0.219	−0.054	–	–	–
PHE36	−1.285	−1.374	0.088	−1.224	−1.142	−0.082
TRP37	–	–	–	−0.878	−0.448	−0.430
ILE52	−1.349	−1.553	0.204	−1.880	−1.700	−0.180
ALA56	−0.383	−0.569	0.185	−0.967	−1.108	0.141
GLN59	−1.109	−0.981	−0.129	–	–	–
LEU61	−1.481	−1.659	0.177	−1.897	−1.906	0.009
VAL62	–	–	–	−1.666	−1.738	0.072
TYR67	–	–	–	−2.049	−1.855	−0.194
ARG68				−0.833	−0.741	−0.092
MET69				−1.262	−1.337	0.075
PHE77	−1.404	−1.783	0.379	−0.061	−0.412	0.473
ILE91	−1.350	−1.260	−0.090	−0.295	−0.264	−0.031
ILE95	−3.348	−3.445	0.097	−2.878	−2.964	0.086
GLU99	–	–	–	−2.575	−2.008	−0.567
ARG111	–	–	–	−1.269	−1.490	0.221
VAL112	−0.515	−0.730	0.215	−1.873	−2.000	0.127
VAL115	−2.391	−2.418	0.027	−3.168	−3.170	0.002
SER116	−2.254	−2.360	0.106	−1.693	−1.941	0.247
PHE119	−3.560	−3.639	0.078	−1.987	−1.940	−0.047
ARG120	−10.096	−2.088	−8.007	–	–	–
LEU135	−0.498	−0.366	−0.132	−0.445	−0.339	−0.106

*E_total_, total interaction energy; E_vdw_, Van der Waals energy; E_eie_, electrostatic interaction energy*.

### Fluorescence displacement binding assay of mutants

**Figure 6:**
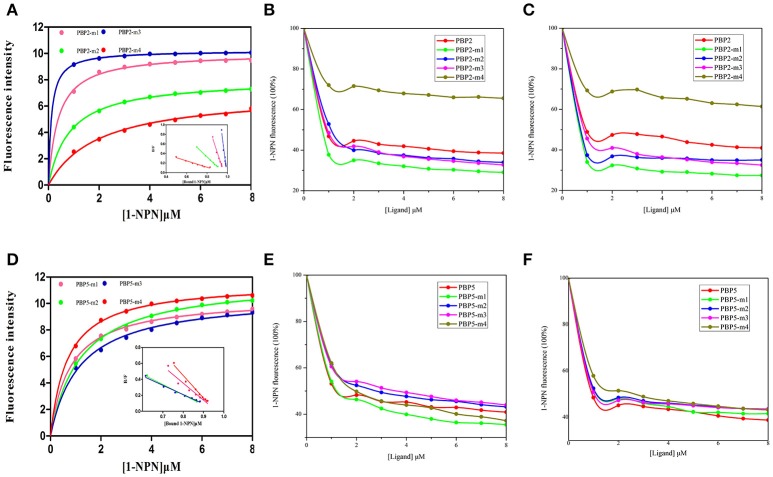
Binding of 1-NPN and ligands to CpunPBP2 and CpunPBP5 mutants. **(A)** The binding curve and relative Scatchard plots of CpunPBP2 and mutants. **(B)** Competitive binding curves of CpunPBP2 and mutants to *E*10-16:Ald. **(C)** Competitive binding curves of CpunPBP2 and mutants to *Z*10-16:Ald. **(D)** The binding curve and relative Scatchard plots of CpunPBP5 and mutants. **(E)** Competitive binding curves of CpunPBP5 and mutants to *E*10-16:Ald. **(F)** Competitive binding curves of CpunPBP5 and mutants to *Z*10-16:Ald.

The recombinant mutant proteins were expressed and purified as described for wild type and analyzed by SDS-PAGE (Figure S3). The emission wave lengths of mutants with 1-NPN were 400–410 nm. The binding curve (Figure 6) of CpunPBP2 and CpunPBP5 mutants with 1-NPN complexes were made. The binding affinities of mutant between proteins and sex pheromones are listed in Table 4. The results showed that, compared with CpunPBP2, the mutant Cpun2-m4 likely lost the binding ability to the two sex pheromones (Figure 6). The binding abilities of the three remaining mutants show no significant differences with wild CpunPBP2. Compared with CpunPBP5, the binding ability of all CpunPBP5 mutants to sex pheromones are reduced by varying degrees (Figure 6). The binding affinity of mutant of CpunPBP5-m3 to *E*10-16:Ald decreased the most, and the binding capacity of CpunPBP5-m4 to *Z*10-16:Ald also decreased considerably.

**Table 4:** IC_50_ values (μM) and calculated dissociation constants (K_D_) (μM) of CpunPBP2 and CpunPBP5 with their mutants to two pheromones.

**Proteins**	***E*****10-16:Ald**		***Z*****10-16:Ald**	
	**IC50**	**K_D_**	**IC50**	**K_D_**
CpunPBP2	0.94 ± 0.03 b	0.40 ± 0.01 b	0.98 ± 0.09 b	0.42 ± 0.04 b
CpunPBP2-m1	0.80 ± 0.02 b	0.22 ± 0.01 b	0.76 ± 0.01 b	0.21 ± 0.00 b
CpunPBP2-m2	1.18 ± 0.20 b	0.54 ± 0.09 b	0.79 ± 0.03 b	0.36 ± 0.01 b
CpunPBP2-m3	1.04 ± 0.13 b	0.11 ± 0.01 b	0.92 ± 0.05 b	0.10 ± 0.00 b
CpunPBP2-m4	30.04 ± 17.59 a	19.93 ± 11.67 a	15.37 ± 3.18 a	10.20 ± 2.11 a
CpunPBP5	1.33 ± 0.06 c	0.60 ± 0.02 c	0.97 ± 0.02 c	0.36 ± 0.01 d
CpunPBP5-m1	1.63 ± 0.01 bc	0.72 ± 0.00 c	1.41 ± 0.02 b	0.72 ± 0.01 c
CpunPBP5-m2	2.73 ± 0.33 a	1.49 ± 0.18 ab	1.66 ± 0.03 b	0.90 ± 0.02 ab
CpunPBP5-m3	3.64 ± 0.10 a	1.85 ± 0.16 a	1.62 ± 0.04 b	0.82 ± 0.02 bc
CpunPBP5-m4	2.61 ± 0.78 ab	1.06 ± 0.32 bc	2.57 ± 0.55 a	1.04 ± 0.22 a

*For each pheromone compound, different letters within a column of each protein indicate significant differences (LSD test, P < 0.05)*.

### Tissues-specific expression pattern of CpunPBP2 and CpunPBP5

**Figure 7:**
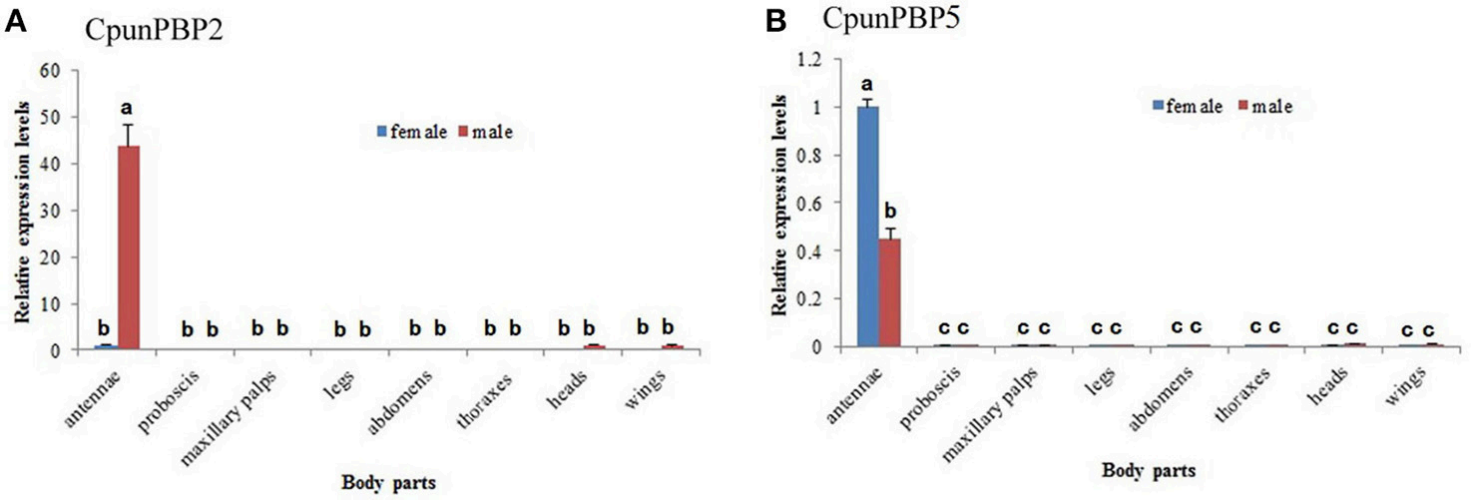
Expression pattern analysis of *CpunPBP2* and *CpunPBP5* in different tissues. **(A)** Expression levels of CpunPBP2 in different tissues. **(B)** Expression levels of CpunPBP5 in different tissues. The different letters (a, b, c) above each bar represented significant differences (*p* < 0.05).

The expression levels of CpunPBP2 and CpunPBP5 in different tissues (male and female antennae, proboscises, maxillary palps, thoraxes, legs, abdomens, heads, and wings) were evaluated using qPCR. The target product was largely amplified in antennae, with low expression level in other tissues (Figure 7). CpunPBP5 is mainly expressed in the female antennae, which contrasts with CpunPBP2 and its male-specific expression. In general, expression levels of CpunPBP2 and CpunPBP5 in proboscises, maxillary palps, thoraxes, legs, abdomens, heads, and wings were very low or null.

## Discussion

Odorant binding proteins are essential for insect olfactory perception because they are transporters between the external environment and insect chemoreceptors (Sun Y. L. et al., 2013). Fluorescence binding affinity has emerged as an important method to demonstrate binding capacity with ligands and help elucidate mechanisms of OBPs (Campanacci et al., 2001; Fan et al., 2011). Jia et al. (2015) cloned a PBP from *C. punctiferalis* and named as CpunPBP1 (GenBank accession number: KP027286), which is similar to CpunPBP2 we obtained. But in 2016 (Jia et al., 2016), they got the same sequence by transcriptome analysis and named as CpunPBP2 (GenBank accession number: GEDO010000019.1). In order to eliminate the confusion, we use the second name in our study. In this study, CpunPBP2 and CpunPBP5 had strong binding abilities with two sex pheromone compounds, indicating that the two PBPs may play important roles in transporting sex pheromones within the sensillar lymph. Furthermore, CpunPBP2 and CpunPBP5 also bind volatiles: 1-tetrodecanol, trans-caryopyllene, farnesene, and β-farnesene, which suggest CpunPBP2 and CpunPBP5, may share similar amino acid binding sites with GOBPs associated with the volatiles (Mao et al., 2016). Interestingly, CpunPBP2 and CpunPBP5 discriminate the chiral structure of chemical molecules, similar to AlinOBP5 results in *Adelphocoris lineolatus* (Wang et al., 2013). We speculate that the chiral structure of ligands may affect the binding constants and need to be further investigated.

Protein structure plays crucial roles in recognition and binding of ligand molecules. Studies of the interactions between proteins and ligands are necessary to better understand the binding mechanism. Structures of OBP and PBP in other lepidopteran insects, such as *B. mori* (Sandler et al., 2000; Horst et al., 2001), *A. polyphemus* (Mohanty et al., 2004) and *A. transitella* (Xu et al., 2010; di Luccio et al., 2013), were used to provide insights into our PBPs. In this study, the key residues were evaluated based on the energy values. After site-directed mutagenesis, four mutants of CpunPBP2 and four mutants of CpunPBP5 protein were purified and used to analyze the binding mechanism. Compared with CpunPBP2, the binding ability of CpunPBP2 mutants were not significantly reduced, expect for CpunPBP2-m4. We speculate that the amino acid substitution of the three mutants of CpunPBP2 had a slight effect of relaxing the compact structure of the binding site, similar to the loss of high specificity with *Plutella xylostella* mutants (Zhu et al., 2016). Because Phe115 in CpunPBP2 had a stronger hydrophobic interaction than other amino acids (Table 2) and the binding affinity between CpunPBP2-m4 and sex pheromone compounds sharply decreased, we speculate that Phe115 in CpunPBP2 are involved in sex pheromone recognition. The binding abilities of CpunPBP5 mutants with sex pheromones varied, which suggests that the small protein modifications may have affected the hydrogen bond between protein and sex pheromones. These results may be due to the change of hydrocarbon interactions or the stabilization of the hydrophobic binding pocket. This suggests that the conformation of PBP was influenced by the transformation of the single amino acid (Zhang et al., 2017). Further research using NMR or x-ray to analyze the protein structure may be necessary to better understand these changes.

The expression levels measured by qPCR showed that *CpunPBP2* and *CpunPBP5* were mainly expressed in antennae, with low expression in the other tissues. These results suggest that CpunPBP2 and CpunPBP5 play a crucial role in odorant chemoreception (including sex pheromone). CpunPBP2 gene was more abundantly expressed in male antennae than in female antennae, which is similar to results found in other insects, including *Spodoptera exigua, P. xylostella, Agrotis ipsilon, Helicoverpa armigera*, and *Maruca vitrata* (Xiu and Dong, 2007; Zhang et al., 2011; Gu et al., 2013; Sun M. J. et al., 2013; Mao et al., 2016). High expression of CpunPBP2 in male antennae may indicate that CpunPBP2 is involved in male-female recognition. Expression level of the CpunPBP5 gene in male antennae was lower than that of female antennae, which is similar to results found with *M. vitrata* and *Sesamia inferens* (Jin et al., 2014; Mao et al., 2016). Thus, these results suggest CpunPBP2 may be involved in the detection of conspecific sex pheromone and autodetection of sex pheromone compounds (Yang et al., 2009; Holdcraft et al., 2016; Mao et al., 2016).

In conclusion, our study provides key information about CpunPBP2 and CpunPBP5 in *C. punctiferalis*, which may be useful for developing effective pest management strategies for this pest.

## Author contributions

TZ and ZW: Conceived and designed the experimental plan; XG: Preformed the experiments; XG, TZ, and SB: Analyzed the sequence and data; ZW and KH: Provided all the materials and lab facilities necessary for this work; TA and ZW: Revised the manuscript. All authors read and approved the final manuscript.

### Conflict of interest statement

The authors declare that the research was conducted in the absence of any commercial or financial relationships that could be construed as a potential conflict of interest.

## Supplementary material

Figure S1 Multiple sequence alignment of CpunPBP2 and CpunPBP5 with other Lepidopteran insect PBPs.

Figure S2 Phylogenetic tree of CpunPBPs amino acid sequences with other 81 PBPs from different insect species.

Figure S3 SDS-PAGE analyses of purified CpunPBP2 and CpunPBP5 with their mutants. M: marker protein. (A)1:CpunPBP2 original protein. (A)2: CpunPBP2-Phe9 mutant (CpunPBP2-m1). (A)3: CpunPBP2-Phe33 mutant (CpunPBP2-m2). (A)4: CpunPBP2-Ser53 mutant (CpunPBP2-m3). (A)5: CpunPBP2-Phe115 mutant (CpunPBP2-m4). (B)1:CpunPBP5 original protein. (B)2: CpunPBP5-Ser9 mutant (CpunPBP5-m1). (B)3: CpunPBP5-Phe12 mutant (CpunPBP5-m2). (B)4: CpunPBP5-Val115 mutant (CpunPBP5-m3). (B)5: CpunPBP5-Arg120 mutant (CpunPBP5-m4).

Table S1 Primers for expression and qPCR.

Table S2 Primers for mutants of CpunPBP2 and CpunPBP5.
