# Supplementary figures and images for: Binding Specificity of Two PBPs in the Yellow Peach Moth Conogethes punctiferalis (Guenée)
Source: Front Physiol. 2018 Apr 3;9:308. doi: 10.3389/fphys.2018.00308 (PMC5891627; doi:10.3389/fphys.2018.00308)

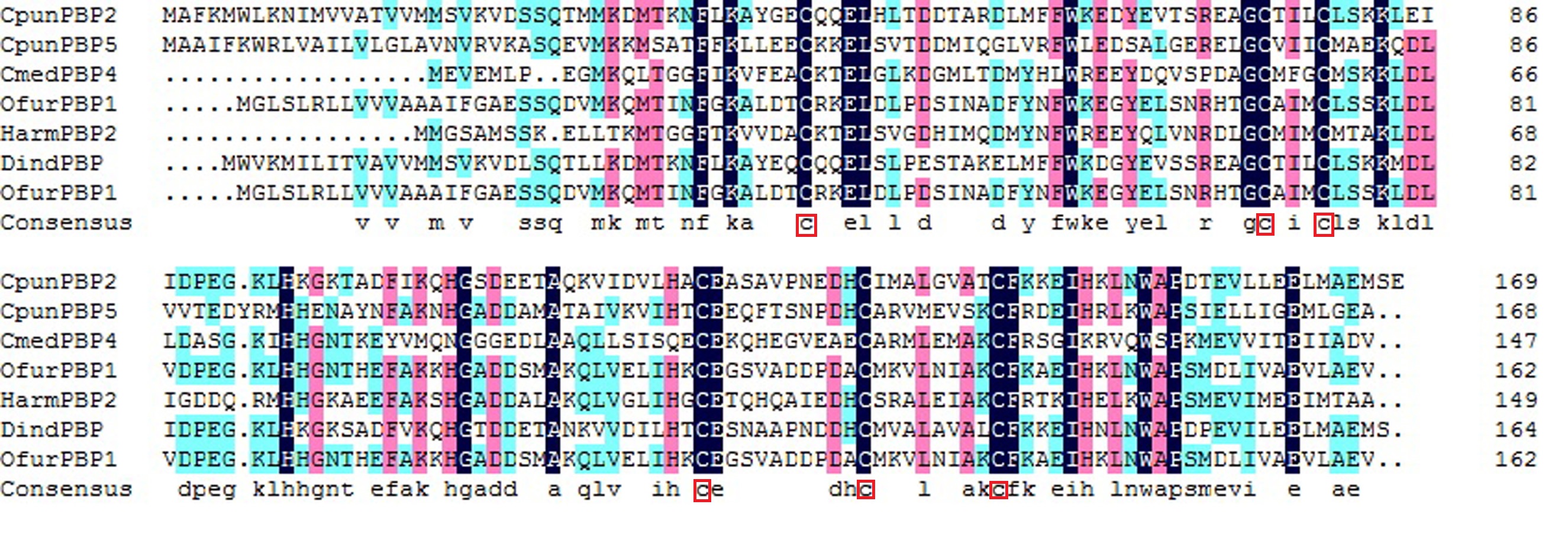

Supplement: Figure S1 — Multiple sequence alignment of CpunPBP2 and CpunPBP5 with other Lepidopteran insect PBPs. [file Image1.jpg]

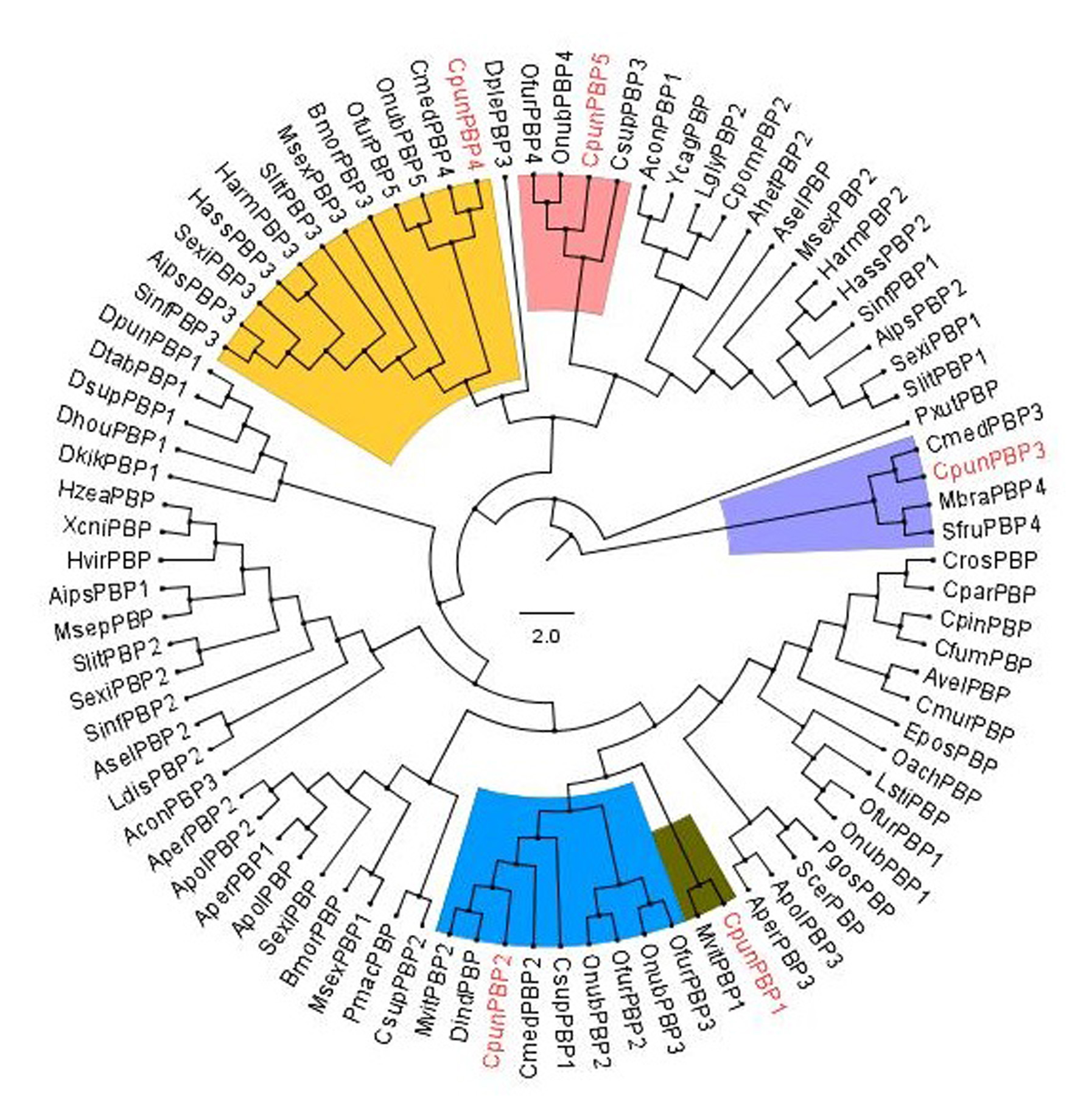

Supplement: Figure S2 — Phylogenetic tree of CpunPBPs amino acid sequences with other 81 PBPs from different insect species. [file Image2.jpg]

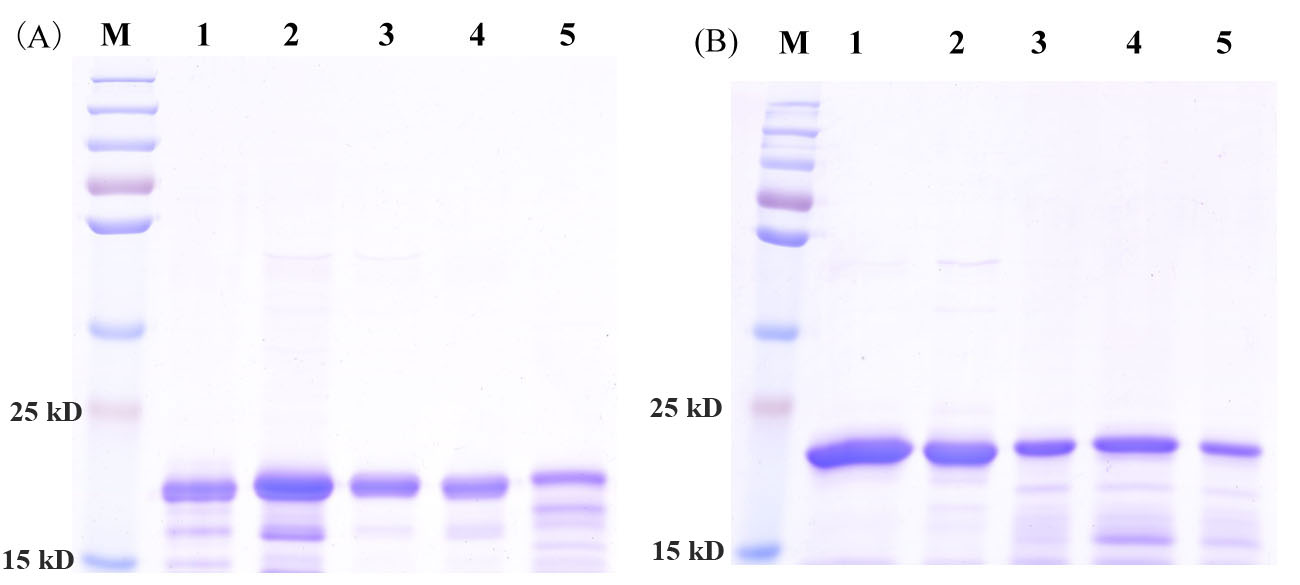

Supplement: Figure S3 — SDS-PAGE analyses of purified CpunPBP2 and CpunPBP5 with their mutants. M: marker protein. (A)1:CpunPBP2 original protein. (A)2: CpunPBP2-Phe9 mutant (CpunPBP2-m1). (A)3: CpunPBP2-Phe33 mutant (CpunPBP2-m2). (A)4: CpunPBP2-Ser53 mutant (CpunPBP2-m3). (A)5: CpunPBP2-Phe115 mutant (CpunPBP2-m4). (B)1:CpunPBP5 original protein. (B)2: CpunPBP5-Ser9 mutant (CpunPBP5-m1). (B)3: CpunPBP5-Phe12 mutant (CpunPBP5-m2). (B)4: CpunPBP5-Val115 mutant (CpunPBP5-m3). (B)5: CpunPBP5-Arg120 mutant (CpunPBP5-m4). [file Image3.jpg]
