# Supplementary material for: Binding Specificity of Two PBPs in the Yellow Peach Moth Conogethes punctiferalis (Guenée)
Source: Front Physiol. 2018 Apr 3;9:308. doi: 10.3389/fphys.2018.00308 (PMC5891627; doi:10.3389/fphys.2018.00308)
Supplement: Table S1 — Primers for expression and qPCR. [file Table1.pdf]

Table S1 Primers for expression and qPCR.

Primers	Primer sequences (5′-3′)
CpunPBP2-F-expression	CTGGATCCATGATGAAGGATATGAC
CpunPBP2-R-expression	CGAAGCTTTCATCGTTCTGACAT
CpunPBP5-F-expression	CTGGATCCTCTCAGGAGGTGAT
CpunPBP5-R-expression	CGAAGCTTCTAGGCTTCTCCTAGC
P2-F-QPCR	GCGGACTTCATCAAGCAAC
P2-R-QPCR	GCCCAGAGCCATTATGC
P5-F-QPCR	GACGGAAGACTACAGGATGC
P5-R-QPCR	GTGAACTGCTCCTCGCATGT
Actin-F-QPCR	CACCCTGAAGTACCCCATCG
Actin-R-QPCR	TGAGGTAGTCGGTCAAGTC
