# Supplementary material for: Binding Specificity of Two PBPs in the Yellow Peach Moth Conogethes punctiferalis (Guenée)
Source: Front Physiol. 2018 Apr 3;9:308. doi: 10.3389/fphys.2018.00308 (PMC5891627; doi:10.3389/fphys.2018.00308)
Supplement: Table S2 — Primers for mutants of CpunPBP2 and CpunPBP5. [file Table2.pdf]

Table S2 Primers for mutants of CpunPBP2 and CpunPBP5

mutants		Primer names	Primer sequences (5′-3′)
CpunPBP2-m1	Phe9(TTT)-TAT	P2m1-F	GAATTATCTGAAGGCGT
		P2m1-R	ACGCCTTCAGATAATTC
CpunPBP2-m2	Phe33(TTC)-TAC	P2m2-F	GTTCTACTGGAAGGAAGAC
		P2m2-R	GTCTTCCTTCCAGTAGAAC
CpunPBP2-m3	Ser53(TCC)-TAC	P2m3-F	CACCATCTTGTGTCTCTAC
		P2m3-R	GTAGAGACACAAGATGGTG
CpunPBP2-m4	Phe115(TTC)-TAC	P2m4-F	CCACCTGCTACAAGAAG
		P2m4-R	CTTCTTGTAGCAGGTGG
CpunPBP5-m1	Ser9(AGC)-ACC	P5m1-F	GAAAATGACCGCTACGT
		P5m1-R	ACGTAGCGGTCATTTTC
CpunPBP5-m2	Phe12(TTT)-TAT	P5m2-F	GAAAATGAGCGCTACGTATT
		P5m2-R	AATACGTAGCGCTCATTTTC
CpunPBP5-m3	Val115(GTG)-GCG	P5m3-F	AGGCGTCCAAGTGCTT
		P5m3-R	AAGCACTTGGACGCCT
CpunPBP5-m4	Arg120(AGA)-AAA	P5m4-F	GCTTCAAAGACGAGATCC
		P5m4-R	GGATCTCGTCTTTGAAGC
